# SpaFlow: a Nextflow pipeline for QC and clustering of MxIF datasets

**DOI:** 10.1093/bioadv/vbaf032

**Published:** 2025-02-14

**Authors:** Brenna C Novotny, Raymond Moore, Lynn Langit, David Haley, Rachel L Maus, Jun Jiang, Caitlin Ward, Ray Guo, Ellen L Goode, Svetomir N Markovic, Chen Wang

**Affiliations:** Department of Quantitative Health Sciences, Mayo Clinic, Rochester, MN 55905, United States; Department of Quantitative Health Sciences, Mayo Clinic, Rochester, MN 55905, United States; Department of Quantitative Health Sciences, Mayo Clinic, Rochester, MN 55905, United States; Department of Quantitative Health Sciences, Mayo Clinic, Rochester, MN 55905, United States; Department of Medical Oncology, Mayo Clinic, Rochester, MN 55905, United States; Department of Quantitative Health Sciences, Mayo Clinic, Rochester, MN 55905, United States; Division of Biostatistics & Health Data Science, University of Minnesota, Minneapolis, MN 55455, United States; Department of Laboratory Medicine and Pathology, Mayo Clinic, Jacksonville, FL 32224, United States; Department of Quantitative Health Sciences, Mayo Clinic, Rochester, MN 55905, United States; Department of Medical Oncology, Mayo Clinic, Rochester, MN 55905, United States; Department of Quantitative Health Sciences, Mayo Clinic, Rochester, MN 55905, United States

## Abstract

**Motivation:**

Multiplex immunofluorescence (MxIF) enables the quantification of multiple protein markers at a single-cell level while preserving spatial information, offering a powerful tool for studying tissue microenvironments. However, the flexibility in MxIF panel design poses challenges in standardizing cell phenotyping.

**Results:**

We present SpaFlow, an efficient, customizable pipeline for unsupervised clustering and classification of MxIF data, implemented using Nextflow. SpaFlow performs quality control, clustering, and postclustering analysis on segmented and quantified MxIF data, facilitating reproducible and scalable analyses across various computing platforms. The SpaFlow pipeline integrates three clustering and classification packages—Seurat, SCIMAP, and CELESTA—each providing unique methodologies for identifying cell types based on phenotypic markers. A novel “meta-clustering” approach condenses clusters across multiple regions of interest into common meta-clusters, streamlining the cell-type identification process in large datasets. SpaFlow’s robust quality control steps, including signal summation and cell density filtering, mitigate artifacts that may impact clustering accuracy. We demonstrate the utility of SpaFlow in a case study involving 297 ovarian tumor cores, where SpaFlow successfully identified biologically meaningful cell populations, including tumor-infiltrating lymphocytes, efficiently and rapidly. Additionally, SpaFlow’s reproducibility is validated using serial tonsil sections, confirming its capability to consistently identify distinctive cell populations across matched ROIs.

**Availability and implementation:**

SpaFlow is freely available with detailed documentation and examples at https://github.com/dimi-lab/SpaFlow.

## 1 Introduction

Multiplex immunofluorescence (MxIF) is a powerful technique that allows the quantification of multiple protein markers at the single-cell level while retaining critical spatial information within tissue sections. Using fluorescently labeled antibody probes, MxIF iteratively stains and images tissue sections, enabling the segmentation of individual cells based on nuclear and cell membrane markers. This process allows for the precise quantification of proteins within each cell through signal intensity, facilitating cell type identification based on specific protein expression signatures. The downstream analysis further examines the spatial relationships between cells within the tissue microenvironment, making MxIF an invaluable tool for studying complex biological patterns.

MxIF offers the flexibility to design custom panels tailored to a wide range of tissues and biological questions. However, this flexibility also presents challenges in standardizing cell phenotyping across different experiments. Unsupervised clustering methods, which are not reliant on specific panel designs, provide a robust solution by allowing cell type identification using any combination of relevant phenotypic markers. These methods serve as a foundation for human-in-the-loop annotation and supervised classification, enabling more precise and consistent analyses.

To address these needs, we developed SpaFlow, a pipeline for unsupervised clustering and classification of MxIF data implemented with Nextflow (Di Tommaso *et al.* 2017). SpaFlow integrates quality control (QC), clustering, and postclustering analysis on previously segmented and quantified MxIF data. Its implementation in Nextflow ensures efficiency, portability across various computing platforms, and the flexibility to incorporate multiple programming languages and analytical packages in a single workflow.

To demonstrate the utility of SpaFlow, we applied it in three distinct case studies. The first case study focuses on a biological application, analyzing a cohort of 297 ovarian tumor cores to explore the tumor immune microenvironment. This case study highlights SpaFlow’s ability to identify and characterize biologically relevant cell populations, such as tumor-infiltrating lymphocytes (TILs), within complex tissue samples. The second case study addresses the technical reproducibility of SpaFlow by analyzing serial sections of tonsil tissue. Here, we designed a replicate study with 20 physically adjacent fields of view (FOVs) per section, assessing the consistency of SpaFlow’s clustering and spatial mapping across these replicates. The third case study demonstrates SpaFlow’s ability to handle different marker panel designs. We identified cell phenotypes in a separate dataset of oropharyngeal squamous cell carcinoma (OPSCC) patients and validated the phenotype accuracy against human annotations. Together, these use cases illustrate SpaFlow’s versatility in both biological research and technical validation, underscoring its value as a comprehensive tool for MxIF data analysis.

## 2 Implementation and key modules

**Figure 1.**
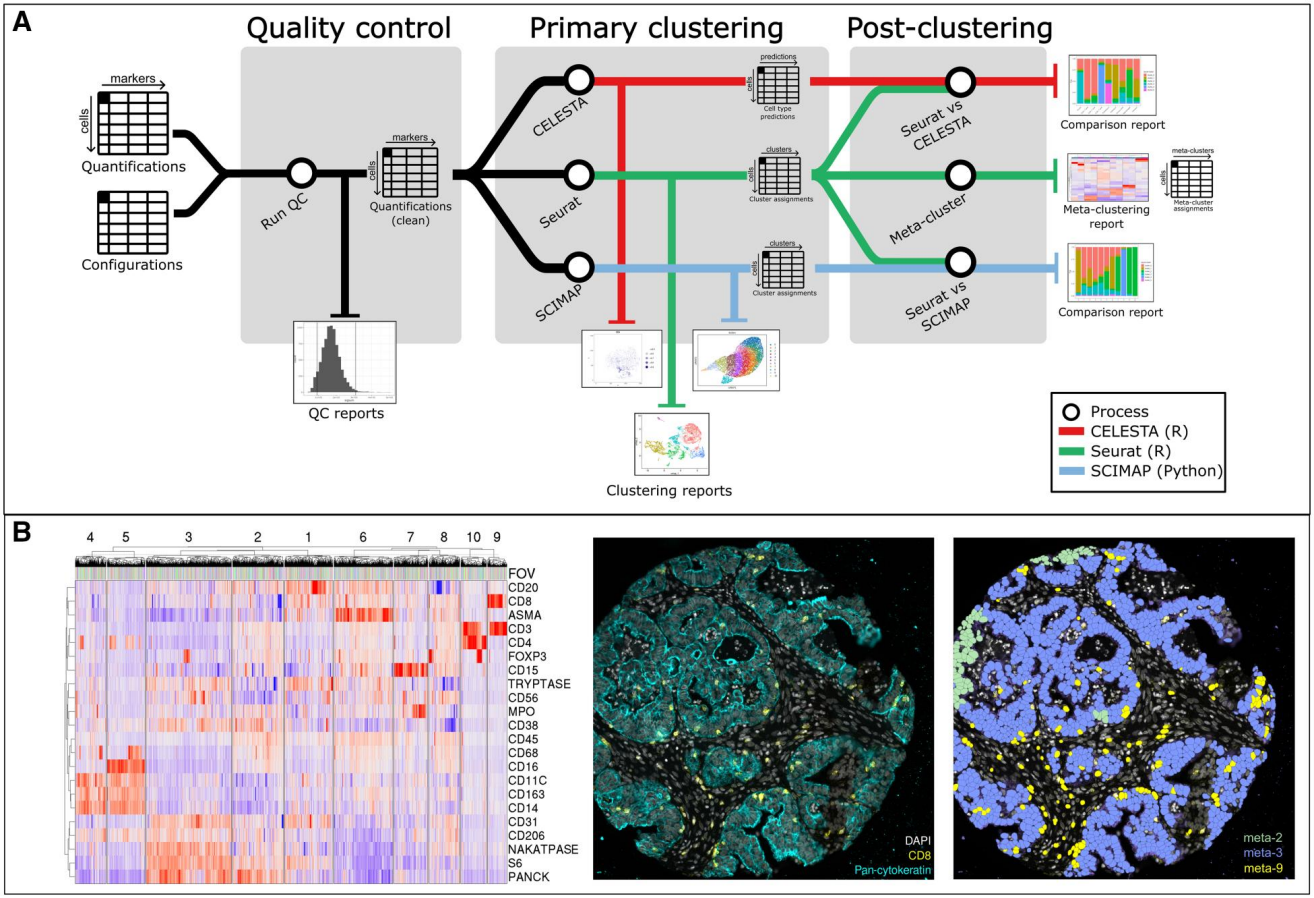
SpaFlow workflow and application in MxIF analysis. (A) Schematic overview of the SpaFlow pipeline. The pipeline begins with the input of raw quantified marker intensities and configuration files, followed by a QC process that outputs cleaned quantification data and QC reports. The primary clustering is then performed using three clustering methods: CELESTA, Seurat, and SCIMAP, each producing clustering reports and cluster assignments. In the postclustering step, SpaFlow integrates the clustering results through meta-clustering, creating a common set of clusters across all FOVs. Comparison reports are generated to evaluate the consistency between the different clustering methods. (B) Example of SpaFlow output applied to MxIF data. The left panel shows a heatmap of marker expression levels across different FOVs, with markers listed on the *y*-axis and FOVs on the *x*-axis. The right panels display representative images of tissue sections, with the left image showing DAPI and pan-cytokeratin staining and the right image illustrating meta-cluster assignments across the tissue section.

Illustrated as Fig. 1A, SpaFlow is implemented as a containerized Nextflow pipeline, deployable on any compatible computing system, such as high-performance computing clusters or cloud providers like Amazon Web Services or Google Cloud Platform. Designed to handle the complexities of MxIF, which involves multiple iterative staining and imaging steps prone to artifacts and errors, SpaFlow requires raw quantified marker intensities per cell as inputs, which can be exported from quantifiers like QuPath (Bankhead *et al.* 2017). The nature of MxIF data generation, including multiple iterations of marker staining and the requirement to segment potentially hundreds of thousands of cells per image, makes it susceptible to artifacts such as areas of high or low marker staining and incorrectly segmented cells. These issues can adversely affect clustering results and interfere with accurate cell phenotyping. SpaFlow addresses these challenges through its highly customizable and configurable options, offering multiple configuration settings. The outputs consist of comprehensive reports and tables suitable for downstream analysis, including cleaned quantification files and mappings of each cell’s coordinates to its assigned cluster or cell type identity across various clustering methods, including a meta-clustering approach. Examples of the SpaFlow outputs as combined FOV heatmap and individual marker layouts in original tissues are shown in Fig. 1B. A full guide to prepare input data files, configuration, and running the pipeline is provided in Supplementary Material Sections S1 and S2, with examples of inputs, outputs, and configurations available on the SpaFlow code repository.

Here we provide a brief overview of the key SpaFlow modules (the detailed interpretations of key output files associated with each module are explained in Supplementary Material Section S3).

### 2.1 Quality control module

SpaFlow performs two QC steps: signal summation and cell density filtering. The signal summation filter checks for abnormally high or low overall fluorescent signal within each cell, which may indicate artifacts or incorrectly segmented cells. The cell density filtering removes extraneous cells by counting the number of cells within a sliding window of a predefined size. Filtering cells with low density eliminates cells which have dissociated from the tissue, or artifactual segments identified outside the tissue. The parameters for all QC steps are configurable within SpaFlow.

### 2.2 Clustering module

SpaFlow provides a wrapper for multiple clustering methods, which facilitates technical convenience and flexibility. SpaFlow also allows for easy parallelization of these clustering methods, which can be vital when working with large datasets. Although many tools exist for clustering of spatial transcriptomics data, fewer are available for MxIF specifically. Therefore, we have chosen packages that cater toward the specific needs of single-cell multiplex-imaging data. SpaFlow employs three clustering and classification packages: Seurat (Hao *et al.* 2024), SCIMAP (Nirmal and Sorger 2024), and CELESTA (Zhang *et al.* 2022). Seurat, originally developed for single-cell RNA-sequencing analysis but later extended to MxIF and other spatial modalities, is an R package that performs dimension reduction and Louvain community detection (Blondel *et al.* 2008) on the cells of each ROI individually. In SpaFlow, the clustering resolution can be set manually, or automatically determined using the *clustree* R package (Zappia and Oshlack 2018). SCIMAP, a Python package designed for spatial data analysis, performs dimension reduction and Leiden community detection (Traag *et al.* 2019). CELESTA is an R package that implements an unsupervised classification algorithm for MxIF, employing a probability-based method to classify cell lineages based on provided matrix of expected marker signatures for each cell type.

### 2.3 Postclustering analysis module

The outputs of each clustering step in SpaFlow are then passed into the postclustering analysis. For the Seurat output, we have developed a “meta-clustering” approach to combine the Seurat clusters from the individual ROIs. This method calculates the centroid (median) marker intensity for each marker in each Seurat cluster and employs hierarchical k-means clustering to create “meta-clusters,” a common set of clusters across all images in the dataset. Each resulting meta-cluster will be composed of similar clusters from different ROIs, allowing them to be classified together. The meta-clustering approach is advantageous in the case of datasets with many ROIs, where there may be thousands of total clusters within the dataset. Arcsine/*Z*-score normalization is performed on the quantification values from individual ROIs before meta-clustering to ensure comparability between ROIs. For the SCIMAP and CELESTA output, SpaFlow creates HTML reports to compare their results to the Seurat results.

## 3 Case studies

As evaluations of SpaFlow, we conducted three case studies with details in Supplementary Material Sections S4–S6.

### 3.1 Case study 1: ovarian cancer tissue microarray

In the first case study, SpaFlow was applied to a tissue microarray (TMA) of ovarian tumor cores, involving 39 markers captured by the IN Cell Analyzer 2500HS (GE Healthcare) platform as described previously (Maus *et al.* 2022). Cells were segmented on DAPI (nucleus) and sodium/potassium ATPase and CD45 (membrane) channels using the DeepCell Mesmer segmentation model (Greenwald *et al.* 2022), resulting in 739 474 cell detections across 297 FOVs. Clusters were generated based on 24 phenotype markers, and the meta-clustering method identified 10 distinct meta-clusters. These meta-clusters were further analyzed in collaboration with a pathologist, leading to the identification of specific cell phenotypes. Notably, meta-clusters meta-2 and meta-3 showed high expression of tumor markers pan-cytokeratin and sodium-potassium ATPase, while meta-9 exhibited high expression of CD3 and CD8, markers characteristic of cytotoxic T cells. The proportion of meta-9 cells correlated significantly with higher CD8 TIL scores from a central pathology review (Goode *et al.* 2017). CD8 TIL score was positively correlated with proportion of cells in meta-9 (Spearman *P*-value <.001).

### 3.2 Case study 2: replication in serial tissue sections

In the second case study, SpaFlow was applied to replicate tonsil samples to evaluate its consistency and robustness in analyzing tissue-specific cell populations. The pipeline successfully identified and replicated key cell phenotypes across multiple tonsil FOVs, demonstrating SpaFlow’s reliability in producing consistent results (e.g. for an example FOV with serial sections of MxIF measures, the correlation coefficient was 0.85 for cellular compositions derived from SpaFlow pipeline). This case study also demonstrates SpaFlow’s utility in identifying large staining artifacts. In one FOV, a large nonspecific staining artifact was present in one technical replicate but absent in the other. A cluster formed consisting entirely of cells affected by this artifact, and the heatmap allowed efficient identification of the marker causing the artifact. This highlights another way SpaFlow can be used in the QC evaluation of MxIF datasets. The key takeaway from this study is that SpaFlow not only efficiently processes large datasets but also maintains high reproducibility across replicates, making it a powerful tool for consistent and accurate spatial biology analysis.

### 3.3 Case study 3: OPSCC

The third case study demonstrates SpaFlow’s flexibility across different panel designs, tissue types, and imaging platforms. We analyzed a dataset of ∼20 000 cells across 64 expert-annotated ROIs from OPSCC (Bartemes *et al.* 2025) generated on the Hyperion multiplex imaging mass cytometry platform (Standard Biotools). Compared to the ovarian TMA acquired in Use Case 1, the OPSCC dataset introduces several markers which are absent in the ovarian dataset, including vimentin, CD11b, CD45RA, and CD45RO. Conversely, the OPSCC panel does not include several markers available in the ovarian dataset, including the isotype controls, tryptase, and MPO. Based on the meta-cluster output with 18 meta-clusters, cells were classified into six phenotypes: B cells, CD4 T cells, CD8 T cells, macrophages, stromal cells, and tumor cells. Manual annotations for the corresponding cell type were enriched in all predicted phenotypes of the meta-cluster solution. Misclassified cells were shown to have higher expression of markers characteristic of the misclassified cell type when compared to their correctly classified counterparts, likely caused by overlapping signal from neighboring cells of the misclassified type. To achieve increased accuracy in these more ambiguous instances, visualization of SpaFlow results in QuPath as described in Supplementary Material Section S3 can facilitate iterative refinements to annotate and train supervised classification models. In conclusion, despite differences in panel design, biologically relevant phenotypes were discerned in both the ovarian and OPSCC studies.

### 3.4 Performance

For all three case studies, we were able to gain these insights quickly: for the first use case, 297 ROIs were processed in 33 min, with a maximum of 1GB memory for a single process. For the second use case, each tonsil’s set of 20 FOVs ran in ∼4 min, with a maximum memory usage of 1.4GB. For the third use case, 64 ROIs were processed in 9 min, with a maximum memory usage of 1GB. All analyses were run with a maximum parallelization of 10 threads per process.

## 4 Discussion

We have developed SpaFlow to address critical challenges in the analysis of MxIF data, particularly in managing the high dimensionality and complexity inherent in this data modality. By leveraging the Nextflow and Docker frameworks, SpaFlow ensures flexibility and scalability across various computational environments, making it accessible to a wide range of users. The inclusion of meta-clustering allows for a more streamlined and consistent identification of biologically meaningful clusters across large datasets, while the automated QC steps enhance data reliability by filtering out potential artifacts. The pipeline’s integration into a human-in-the-loop process further enables iterative refinement and validation, ensuring that the final classifications are both accurate and interpretable. Looking forward, expanding the pipeline to incorporate supervised classification methods will further enhance its utility, providing users with more robust tools for comprehensive spatial biology analyses.

## Supplementary Material

vbaf032_Supplementary_Data

## Data Availability

The data underlying this article are available in ImmunoAtlas, at https://immunoatlas.org/MYCB/240802-1/MYCB24004/. The code of SpaFlow is freely available under MIT license at https://github.com/dimi-lab/spaflow.
