# Supplementary material for: SpaFlow: a Nextflow pipeline for QC and clustering of MxIF datasets
Source: Bioinform Adv. 2025 Feb 14;5(1):vbaf032. doi: 10.1093/bioadv/vbaf032 (PMC11879158; doi:10.1093/bioadv/vbaf032)
Supplement: vbaf032_Supplementary_Data [file vbaf032_supplementary_data.zip › Supplementary Materials.pdf]

Supplementary Materials

Contents

Section-1: Input files and configuration 2
Input files 2
Configuration options 2
Section-2: Running the SpaFlow Pipeline 4
Section-3: Interpretation of SpaFlow Results 6
QC modules 6
A. SigSum report 6
B. Bin density report 6
C. QC-annotated quantification tables 7
Clustering Modules 7
A. Seurat clustering 7
i. Clustering Report 7
ii. Seurat cluster assignments tables 8
B. CELESTA 9
i. CELESTA classification report 9
ii. CELESTA cell classification assignment tables 10
C. SCIMAP 10
i. SCIMAP Clustering Report 10
ii. SCIMAP cluster assignment tables 10
Post-clustering Modules 10
A. Meta-clustering 10
i. Reports 10
ii. Meta-cluster assignment tables 11
B. Comparison reports 12
Exporting intermediate quantification files 12
Loading clusters into QuPath 13
Section-4: Use case 1. Applying SpaFlow for Ovarian Tumor Microenvironment Studies 16
Study motivations 16
Use of SpaFlow and Analytical steps 16
Key Results 17
Section-5: Use Case 2. Reproducibility Assessment of SpaFlow in Serial Tonsil Sections 19
Study motivations 19
Analytical steps and Key Results 19
Section-6: Use Case 3. Assessment of SpaFlow with Alternate Panel Designs with Oropharyngeal Squamous Cell Carcinoma Imaging Mass Cytometry Dataset 21
Study motivations 21
Analytical steps and Key Results 21

Section-1: Input files and configuration

In this section, we will provide an overview of input files, file formats, and configuration options for SpaFlow.

Input files

The inputs to SpaFlow are cell by marker “quantification files”, where each row represents one cell segment, and each column represents a measurement, or quantification, of each marker’s signal within that cell.

The input file format is derived from QuPath marker quantification. Images and cell segmentations should be loaded into QuPath, and measurements calculated for each segmentation object. A script for exporting quantification files from QuPath (export_individual_qupath_rois.groovy) is included in the SpaFlow GitHub repository: https://github.com/dimi-lab/SpaFlow/tree/main

Configuration options

Two configuration files need to be set up: nextflow.config, and, if CELESTA is to be run, celesta_prior_matrix.csv. Templates for these files can be found in the SpaFlow GitHub repository.

nextflow.config

This file specifies the configurations for QC filtering, clustering resolutions, markers to be included for clustering, and run parameters.

Field	Description
celesta_prior_matrix	Path to CELESTA prior matrix file, if running CELESTA
input_dir	Path to directory where "output_tables" and "output_figures" directories should be created
data_pattern	Regex for file extension of input files; default is to accept both CSV and TSV files.
output_dir	Path to directory where output_reports and output_tables directories should be created
qc_only	Run only QC steps. It is advised to run the QC alone first and check results before proceeding with clustering analysis.
run_scimap	Run SCIMAP Leiden clustering?
run_celesta	Run CELESTA classification?
run_seurat	Run Seurat clustering and meta-clustering?
export_intermediates	Create a new directory, "intermediates", in your outputs directory, containing the centroids of the Seurat clusters from each FOV using the CLR normalization method, and those using the arcsine/Z-score method (AKA the inputs for meta-clustering)
sigsum_quantile_high	Upper quantile cutoff for signal summation filtering (default 0.99)
sigsum_quantile_low	Lower quantile cutoff for signal summation filtering (default 0.05)
bin_size	Size of bounding box for low-density cell search (default 50). Smaller bin size is more stringent (will remove more cells at the same density cutoff).
density_cutoff	Cutoff for number of cells within the bounding box defined as low-density (default 5). Higher density cutoff is more stringent (will remove more cells at the same bin size).

cluster_metric	Intensity measurement type (per cell) to use for Seurat clustering (default Median)
clustering_res	If specified, this clustering resolution will be used for all ROIs for the Seurat clustering and will override the Clustree method. Set to NA or remove this row to use the Clustree method. Higher clustering resolutions will create more clusters. (Default NA)
min_res	Minimum clustering resolution to search with Clustree (default 0.1)
max_res	Maximum clustering resolution to search with Clustree (default 1.9)
res_step	Increment for searching clustering resolutions; functions as “by” argument in R function seq() (default 0.2)
min_clusters	Minimum number of clusters for individual ROI clustering in Seurat (default 6)
scimap_resolution	Resolution to be used in SCIMAP’s Leiden clustering function (default 0.5).
min_metaclusters	Starting number of metaclusters to create (default 5)
max_metaclusters	Ending number of metaclusters to create (default 10)
markers	Comma-separated list of markers in the dataset that should be used for clustering. Make sure marker names are formatted identically as they appear in the header of your quantification files.

If markers are not provided or if all markers provided are not present in the dataset, the quantification file will search for a default list of markers; if all default markers are not present, all markers in the dataset will be used, excluding DAPI.

celesta_prior_matrix.csv (optional)

If you would like to run CELESTA, set up the CELESTA prior matrix file according to the CELESTA documentation: https://github.com/plevritis-lab/CELESTA. Two additional columns are needed for SpaFlow: “anchor” and “index”. As per the CELESTA documentation: “The two vectors are required for defining the “high_expression_threshold”, one for anchor cells and one for index cells (non-anchor cells). The thresholds defined how much the marker expression probability is in order to be considered as expressed.” The defaults for CELESTA are 0.9 for anchor thresholds, and 0.5 for index thresholds. These may be adjusted based on the individual markers’ expression probability plots, which are included in the SpaFlow CELESTA reports (see Supplementary Material 3).

Section-2: Running the SpaFlow Pipeline

In this section, we provide major steps for users to clone, install, and test-run SpaFlow. The source code can be found at https://github.com/dimi-lab/SpaFlow/

1. Clone repository to your machine: `git clone https://github.com/dimi-lab/SpaFlow.git`
2. Install all software and package dependencies (see SpaFlow repository) or, for easier dependency management, set up the Docker container and use the Docker profile as specified in the “deployment” folder of the SpaFlow repository.

Manual Installation:

- Most R dependencies may be installed from CRAN with install_packages(), e.g.:

```
install_packages(“ggplot2”)
```

- CELESTA must be installed via devtools::install_github():

```
# install.packages("devtools")
devtools::install_github("plevritis/CELESTA")
```

- ComplexHeatmap must be installed through Bioconductor:

```
if (!requireNamespace("BiocManager", quietly=TRUE))
    install.packages("BiocManager")
BiocManager::install("ComplexHeatmap")
```

- SCIMAP must be installed into a Python virtual environment:

```
conda create --name scimap python=3.10
conda activate scimap
pip install scimap
```

Docker installation (see SpaFlow repository for full instructions):

- Install Nextflow and Docker, and build the image:

```
docker build -f deployment/Dockerfile . -t spaflow:latest
```

3. Place quantification files in your input directory

   i. File names should be in the format <fov_name>.tsv or <fov_name>.csv (e.g. region_001.tsv) – see Section 1 for formatting

4. Set up nextflow.config (and celesta_prior_matrix.csv if running CELESTA). See Section 1 for setup instructions.
5. Call main pipeline script:

   Running locally:

```
nextflow run main.nf -c nextflow.config
```

   Running with Docker:

```
nextflow run main.nf -c nextflow.config -profile docker
```

Example of command line output after a successful run on 20 FOVs:

```
N E X T F L O W  ~  version 23.04.2
Launching `../../main.nf` [modest_brazil] DSL2 - revision: be1f013a7f
executor >  local (145)
[8c/3ad2b2] process > WRITEMARKERFILE     [100%] 1 of 1 ✔
[22/6532be] process > WRITECONFIGFILE     [100%] 1 of 1 ✔
[7f/496446] process > RUNQC (9)           [100%] 20 of 20 ✔
[6e/f575a8] process > COLLECTBINDENSITY   [100%] 1 of 1 ✔
[5c/1fd351] process > COLLECTSIGSUM       [100%] 1 of 1 ✔
[9c/d95ddc] process > RUNSEURAT (12)      [100%] 20 of 20 ✔
[7a/90222f] process > RUNMETACLUSTERS     [100%] 1 of 1 ✔
[66/469f69] process > RUNCELESTA (20)     [100%] 20 of 20 ✔
[18/552af0] process > SEURATVCELESTA (20) [100%] 20 of 20 ✔
[61/758bd9] process > RUNSCIMAP (20)      [100%] 20 of 20 ✔
[f8/fba4fb] process > SCIMAPREPORT (20)   [100%] 20 of 20 ✔
[6c/ca8db1] process > SEURATVSCIMAP (20)  [100%] 20 of 20 ✔
Completed at: 04-Sep-2024 13:27:13
Duration    : 4m 20s
CPU hours   : 1.4
Succeeded   : 145
```

Section-3: Interpretation of SpaFlow Results

Below, we will explain key results from each of the (A) QC, (B) clustering, and (C) post-clustering modules which will be generated as one or more HTML reports.

QC modules

The QC modules flag cells that do not pass the specified QC thresholds. **It is recommended to first run SpaFlow with the option --qc_only to view QC metrics before proceeding with clustering.**

A. SigSum report

Output location: `output_reports/qc/sigsum_report.html`

Cells with consistently high or low signal across all markers may be caused by staining artifacts. The Signal Summation (SigSum) metric calculates the sum of mean channel intensity per cell and flag cells above or below a specified quantile cutoff.

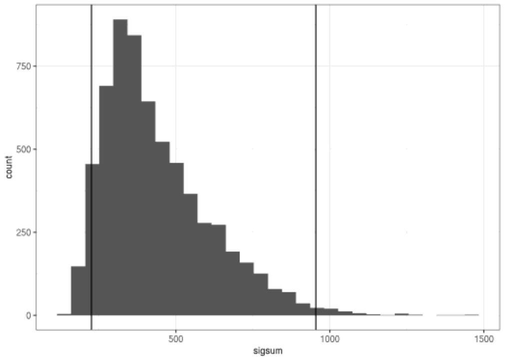

Note: this step flags a set number of cells based on the number of cells within the image. Check the histogram to ensure that the outlying cells are being captured by the specified parameter values.

In configs.csv, sigsum_quantile_high specifies the cutoff above which cells will be flagged for high signal, and sigsum_quantile_low specifies the threshold below which cells will be flagged for low signal.

In the example histogram at right, the SigSum threshold may be too conservative, as cells in the main distribution have been flagged. Examples of appropriate SigSum implementation are shown in the image exmples at right, where red outlined cells were excluded based on low signal sum and high signal sum, indicating improper segmentation and staining artifacts, respectively.

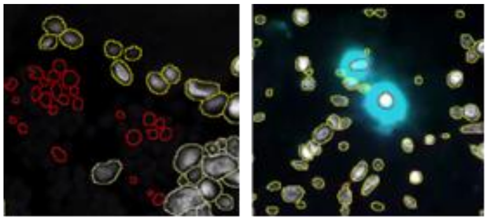

B. Bin density report

Output location: `output_reports/qc/bin_density_report.html`

The bin density metric counts cells in a sliding window of specified dimensions and flags low density regions where cells are too sparse to maintain meaningful spatial relationships.

In configs.csv, bin_size specifies how large the sliding window is (default 50x50 pixels) and density_cutoff specifies the minimum number of cells the window should contain. Any window with fewer than the specified number of cells will flag all cells within the window as low density (default 5 cells). To skip the bin density QC check, specify density_cutoff=0.

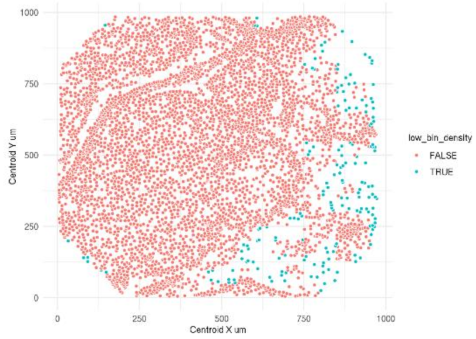

Different datasets may require different windows and/or cutoffs. Be sure to check QC reports to assess whether the specified parameters are appropriate

Example at right of appropriate cell density flagging; cells located far from the main tissue section have been flagged and will be excluded from further analysis.

C. QC-annotated quantification tables

Output location: `output_tables/qc/all_markers_clean_<fov>.csv`

These files are in the same format as the input quantification files, with one row per cell segment, and one column per intensity measurement metric. Additional columns sigsum_metric, sigsum, qc, bin_density, and low_bin_density are added for later filtering in the clustering scripts.

Column	Interpretation
sigsum_metric	Was the cell flagged for having a low or high signal summation? Possible values: “SIGSUM Low” = signal sum was below threshold, “SIGSUM High” = signal sum was above threshold, “Cell” = signal sum was within acceptable limits.
sigsum	Sum of all marker signals, used to determine sigsum_metric based on the specified thresholds.
qc	Did the cell pass QC? Possible values: “Artifact” (cell failed QC and will be removed for clustering steps), “Cell” (cell passed QC)
bin_density	How many cells were in the same window of size specified in the configuration file? Cells in windows with less than the threshold set in the configuration file will be filtered out.
low_bin_density	Was this cell in a window with low cell density? If TRUE, cell will be filtered out.

Clustering Modules

A. Seurat clustering

The Seurat clustering module performs Louvain community detection on each FOV individually.

i. Clustering Report

Output location: `output_reports/seurat/seurat_report_<fov>.html`

Clustree

Cluster resolution is automatically selected based on the SC3 stability metric in the Clustree package. By default, resolutions between 0.1 and 1.9 are searched. The search range can be modified in nextflow.config (see Section 1).

See also: [Zappia L, Oshlack A. Clustering trees: a visualization for evaluating clusterings at multiple resolutions. Gigascience. 2018;7. DOI:gigascience/giy083]

Louvain clustering

Per-ROI clustering is performed on the median pixel intensity per cell. Clustering is based on the Seurat v4 spatial clustering pipeline. Normalization is performed using the CLR method.

UMAP results may not show highly separated clusters; this is normal. When comparing the UMAP representation and the spatial distribution of the clusters (example below), clusters should show some spatial grouping.

See also: https://satijalab.org/seurat/articles/spatial_vignette_2.html

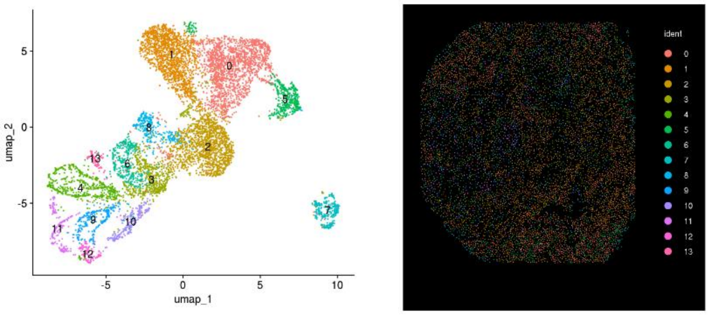

Heatmaps

- Heatmaps are provided for interpretation of cluster composition based on average marker intensity within the cluster.
- There are two sets of heatmaps: “Phenotype markers”, which contains only markers used for clustering/phenotyping, and “All available markers” which displays all markers in the panel.
- Each set contains two heatmaps: “scaled by row” and “scaled by column”. The first is color scaled by row, meaning that the color intensity is relative to the distribution of intensities of a marker across all clusters. The second is scaled by column, meaning that color intensities are relative to the intensities of all markers within a cluster.
- A bright red color in the “scaled by row” plot means that the signal for a given marker is high in that cluster compared to the signal from the same marker in other clusters. A bright red in the “scaled by column” plot indicates that the signal for a marker is high in that cluster compared to other markers in the same cluster. Overall, you can gather similar information from both plots, but they provide useful context for each other.
- Clusters can be given labels based on their expression of characteristic markers for a particular cell type. Clusters should ideally show distinct marker signatures based on the cell type composition of each cluster. Keep in mind that if markers with expression patterns not specifically related to cell type (such as markers related to cell cycle or functional state) are included in the clustering markers, any non-cell type-specific expression of these markers may cause incorrect separation of cell phenotypes.

Example interpretation:

In the below heatmaps, in cluster 4, we see increased expression of tumor markers Keratin 14 and PanCK relative to other markers in cluster 4, and relative to other clusters’ expression of those two markers.

Therefore, we may reasonably label this cluster as “Tumor”. This assignment can be verified on the image in later steps.

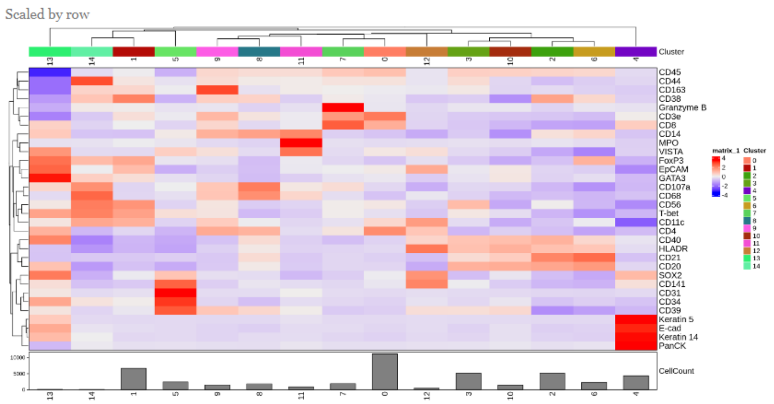

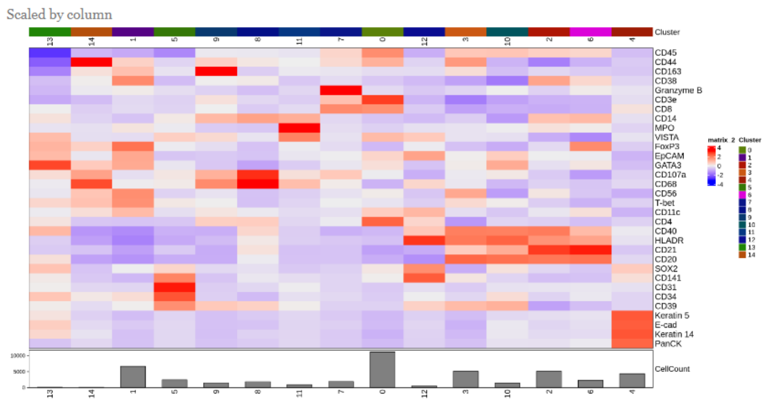

ii. Seurat cluster assignments tables

Output location: `output_tables/seurat/seurat_clusters_<fov>.csv`

One table per FOV is output with the x and y coordinates of each cell in the FOV along with its cluster assignment. Cells that were not included in clustering will show “Artifact” in the seurat_clusters column. These clusters can be visualized on top of the original image with QuPath (see “Loading clusters into QuPath” section below).

B. CELESTA

The CELESTA module uses a probabilistic model to predict the cell types in each FOV individually, based on the expected marker expression profile for each cell type.

i. CELESTA classification report

Output location: `output_reports/celesta/celesta_report_<fov>.html`

For a full explanation of CELESTA outputs, refer to the CELESTA documentation.

Main outputs provided in the SpaFlow CELESTA report include the output of PlotExpProb() for each marker in the matrix to assist with threshold determination according to the CELESTA documentation:

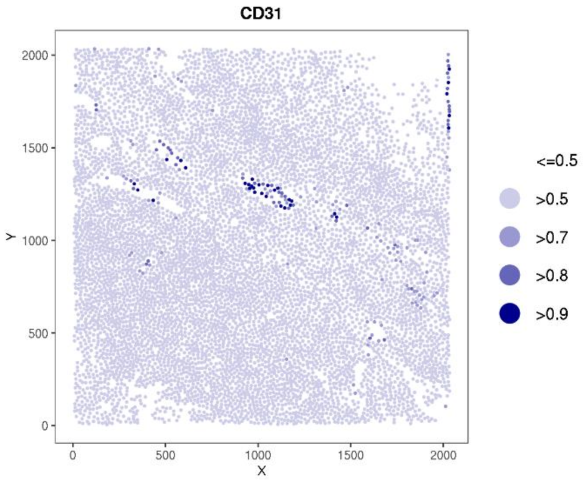

A table of the final cell phenotype assignments by CELESTA:

Cell Type <fct>	Freq <int>
Unknown	652
immune cells	5472
stroma	1093
tumor cells	133
vasculature	726

5 rows

And the output of PlotCellsAnyCombination() to show the final phenotypes assigned by CELESTA:

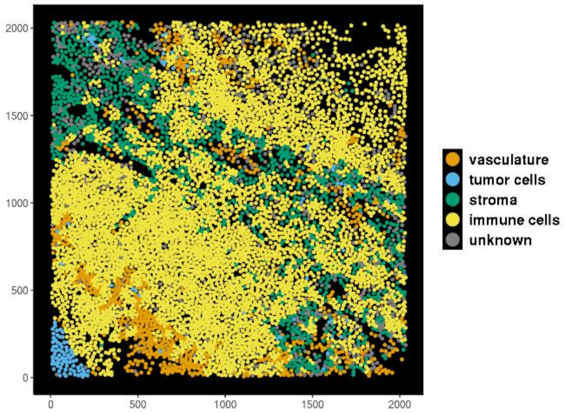

ii. CELESTA cell classification assignment tables

Output location: `output_tables/celesta/celesta_classes_<fov>.csv`

For each cell, x and y coordinates, along with the CELESTA cell type assignment for each round of classification. Any round of classification can be visualized on top of the original image with QuPath (see “Loading clusters into QuPath” section below).

C. SCIMAP

The SCIMAP module performs Louvain community detection on the cells of individual FOVs.

i. SCIMAP Clustering Report

Output location: `output_reports/scimap/scimap_report_<fov>.html`

Similar to the Seurat report, the SCIMAP report includes a UMAP plot of the Leiden clusters, a spatial arrangement plot of the Leiden clusters, and a heatmap of marker expression by cluster. The interpretation of the SCIMAP cluster heatmap follows the same principles as the Seurat heatmaps.

ii. SCIMAP cluster assignment tables

Output location: `output_tables/scimap/scimap_clusters_<fov>.csv`

For each cell, x and y coordinates, along with the SCIMAP cluster assigned to the give cell. The clusters can be visualized on top of the original image with QuPath (see “Loading clusters into QuPath” section below).

Post-clustering Modules

A. Meta-clustering

The meta-clustering module calculates the centroids of the Seurat clusters for each FOV and performs k-means clustering of all Seurat clusters from all FOVs.

i. Reports

Output location: `output_reports/metacluster/seurat_metacluster_report.html`

- k-means clustering of the cluster centroids is performed using the ComplexHeatmap package.

- The meta-cluster heatmaps can be interpreted similarly to the cluster heatmaps, where columns represent the original Seurat clustering output from the previous step. Color intensity represents the scaled mean intensity for each marker (rows).

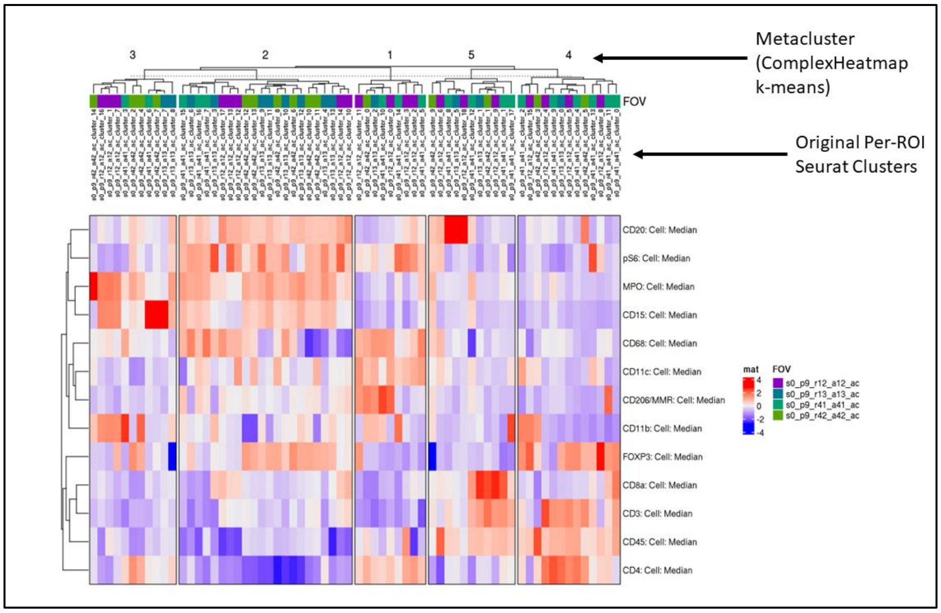

- The original clusters are listed as an annotation above each column, and meta-cluster assignments are located above the dendrogram.
- Meta-cluster heatmaps and mapped coordinate files will be produced for each number of meta-clusters in the range specified in configs.csv. A larger number of meta-clusters will capture more specific expression profiles but require more annotation assignments. You do not need to annotate every version, only the one(s) which you feel best separate the cell types of interest.
- Bar plots representing the proportion of each meta-cluster occupied by each ROI are provided to validate that batch effects are not causing meta-clusters to be segregated by ROI.

At left, an example of appropriate distribution of ROIs across meta-clusters (no meta-cluster is heavily skewed towards one ROI). At right, an example of poor distribution of ROIs across meta-clusters (many clusters show a high bias towards one ROI).

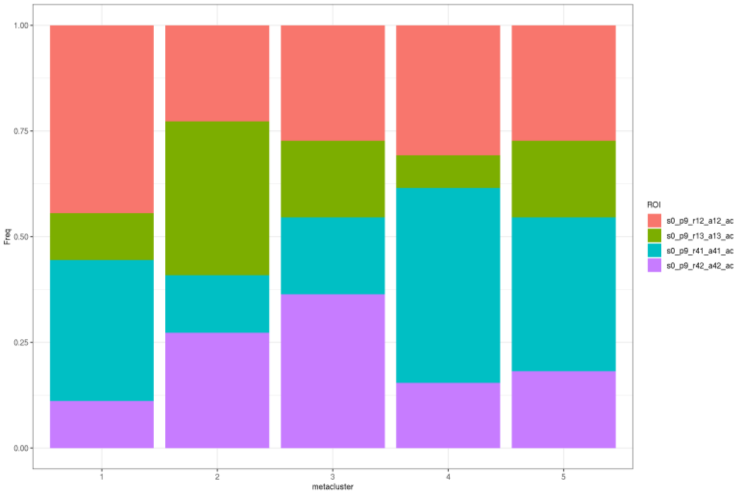

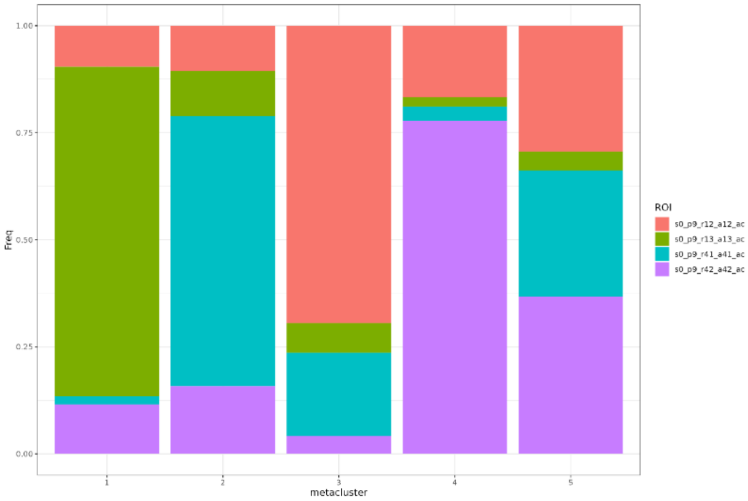

ii. Meta-cluster assignment tables

Output location: output_tables/metacluster/seurat_metaclusters_<fov>.csv

For each cell, x and y coordinates, along with the original per-FOV Seurat cluster and the meta-cluster assignment for each iteration of meta-clustering. The meta-clusters can be visualized on top of the original image with QuPath (see "Loading Clusters Into QuPath" section below).

B. Comparison reports

Output location: `output_reports/seuratvcelesta/seurat_vs_celesta_report_<fov>.html` and `output_reports/seuratvscimap/seurat_vs_scimap_report_<fov>.html`

Comparison reports are generated to compare the clusters generated by Seurat to the clusters generated by SCIMAP and the phenotypes assigned by CELESTA. The reports follow the same structure for each comparison:

First, a table of how many cells are in common between each Seurat cluster and each SCIMAP cluster or CELESTA classification:

	cluster_0	cluster_1	cluster_2	cluster_3	cluster_4	cluster_5	cluster_6	cluster_7	cluster_8	cluster_9
Unknown	75	246	68	79	34	27	32	75	0	4
immune cells	1718	1086	1439	1147	376	695	392	303	319	66
stroma	18	5	1	54	9	7	0	0	0	11
tumor cells	108	170	132	55	1	2	15	6	0	1
vasculature	26	237	65	17	418	1	40	0	10	0

Next, a stacked bar plot showing the proportion of Seurat clusters within each SCIMAP cluster or CELESTA classification:

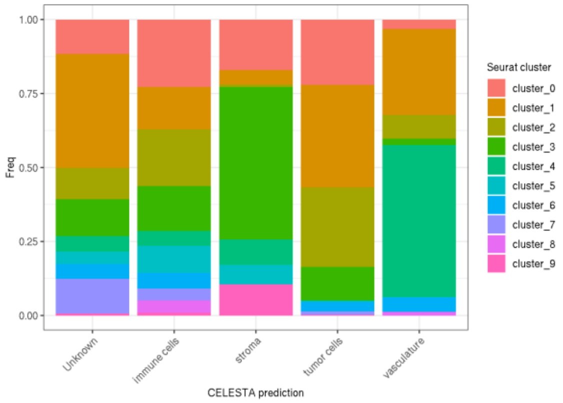

Finally, spatial plots comparing the spatial distribution of the Seurat clusters to the distribution of CELESTA classifications or SCIMAP clusters:

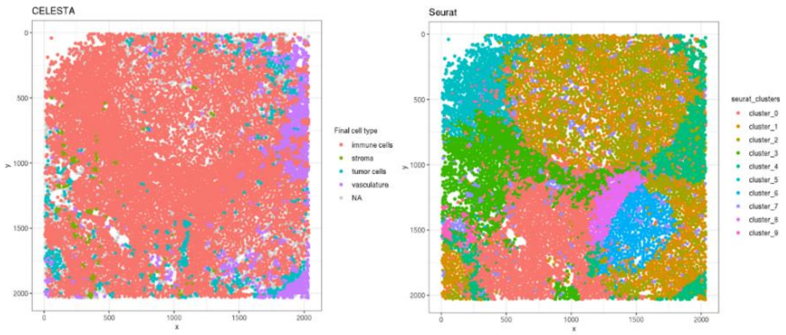

Exporting intermediate quantification files

If the parameter "export_intermediates" is set to "True" in the nextflow.config file (see Supplementary Material 1), a new "intermediates" output directory will be created in output_tables, containing the centroids of the Seurat clusters from each FOV using the CLR normalization method (CLR_seurat_centroids_<fov>.csv), and those using the arcsine/Z-score method (arcsin_zscore_seurat_centroids.csv), which are the inputs for meta-clustering.

Loading clusters into QuPath

Clusters can be visualized in QuPath using the ClusterLabelSwapping.groovy script from the SpaFlow repository. Cluster labels will be overlaid onto the image to verify cluster identities based on marker signal distribution.

1. Open QuPath project
2. Open (double click) the image you would like to view
3. Open the script editor with Automate » show script editor

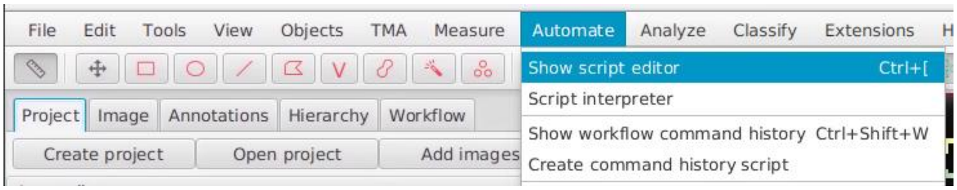

4. Navigate to and open the ClusterLabelSwapping.groovy script with File » Open

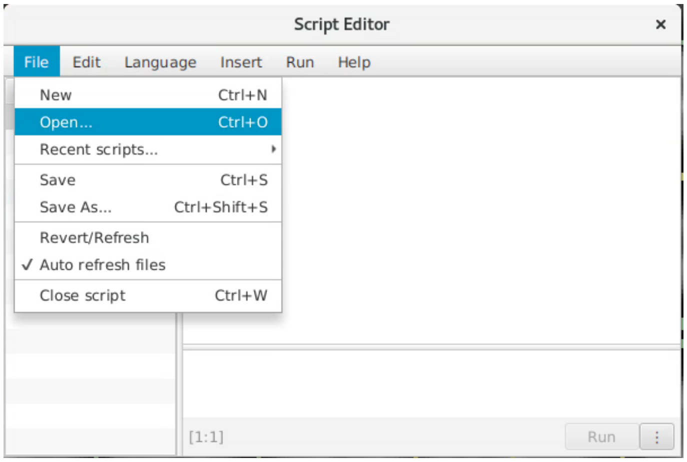

5. Click "Run" in the Script Editor

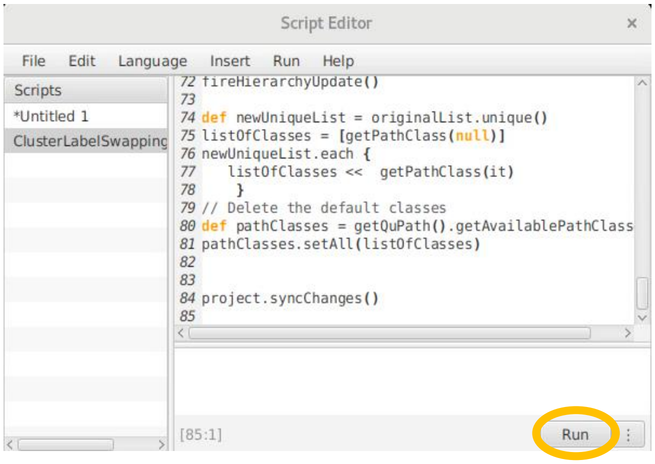

6. File Explorer will open. Navigate to output_tables and select and open the cluster or meta-cluster file that corresponds with the image opened in step 2.

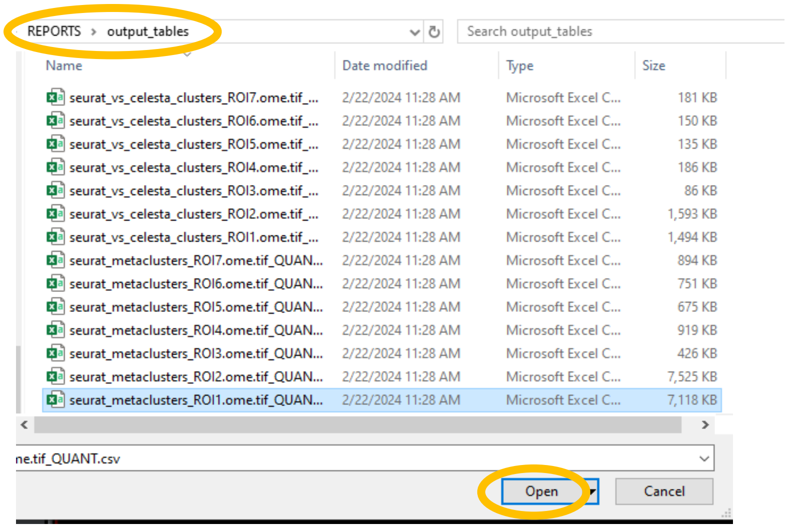

7. A dialogue box will open asking you to choose the column of the file containing the cluster label. Select the label you would like to apply to the image and hit OK.

Note: In the meta-clustering output file, the individual Seurat clusters are in the seurat_clusters column. There are separate columns for each iteration of meta-clustering, labeled with the number of meta-clusters in that iteration.

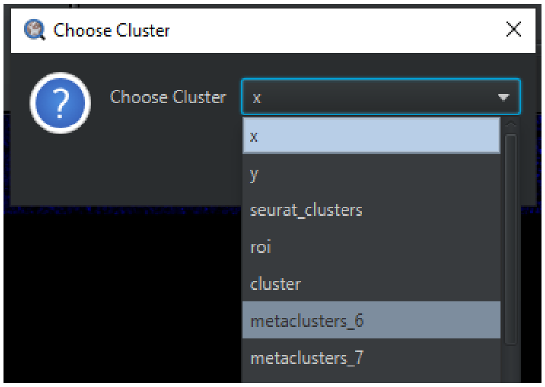

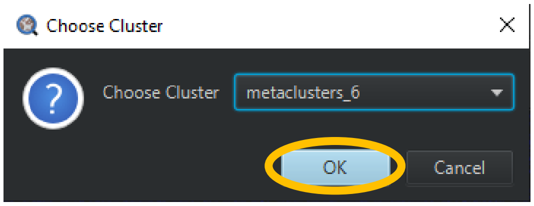

When the clusters load, make sure the cell outlines are turned on. Each cluster will be assigned a color. The cluster assignment for any cell will be shown in the bottom-right of the screen when you mouse over a particular cell.

Turn marker channels on and off with the half-moon brightness/contrast button.

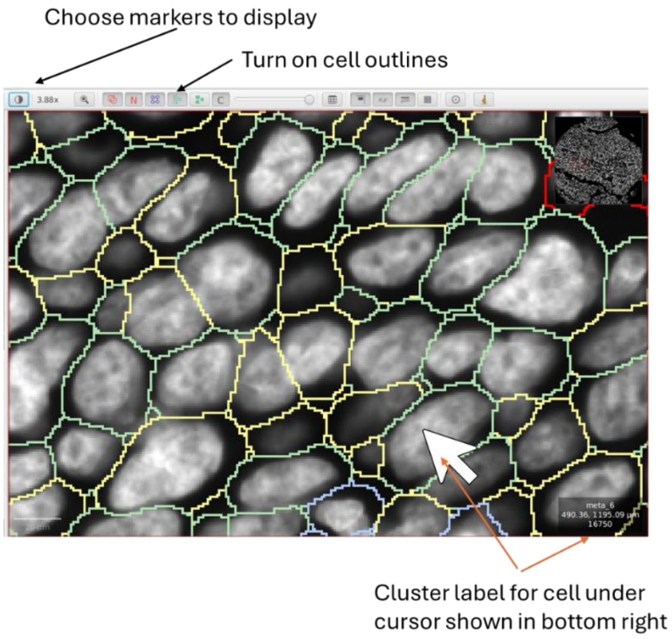

Cluster colors may be changed by double-clicking the cluster label in the Annotations menu.

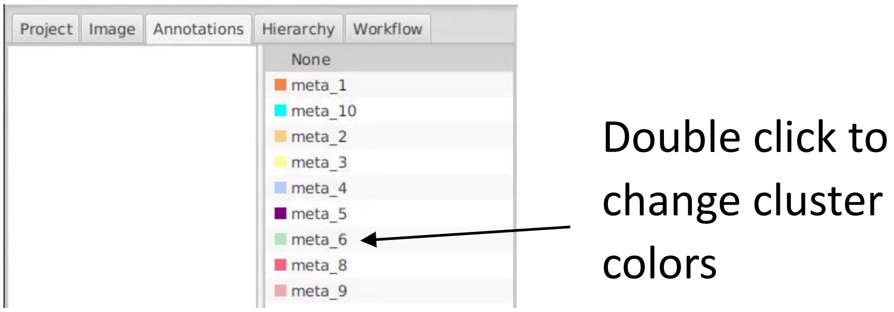

Clusters may be hidden or shown by right-clicking the cluster label » Show/Hide… » Show classes in viewer/Hide classes in viewer.

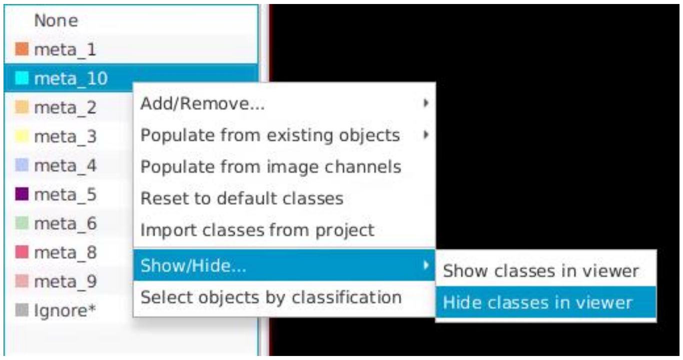

Section-4: Use case 1. Applying SpaFlow for Ovarian Tumor Microenvironment Studies

Study motivations

The tumor immune microenvironment (TIME) plays a crucial role in the progression and treatment response of ovarian cancer. Understanding the spatial distribution and phenotypic characteristics of immune cells within the tumor microenvironment is essential for developing effective therapeutic strategies. In this study, we demonstrate the utility of SpaFlow in (i) analyzing a tissue microarray (TMA) cohort of ovarian tumors, and (ii) categorizing cellular compositions of TIME, and (iii) validating our findings against immunohistochemistry (IHC)-based evaluations of CD8+ tumor-infiltrating lymphocytes (TILs).

Use of SpaFlow and Analytical steps

Our use of SpaFlow began with the input of segmented and quantified cell data files from the ovarian TMA cores (supplementary Fig. 1). The use case demonstrated the logical and technical workflow for analyzing MxIF data: Starting with the input of segmented and quantified cell data files. These data files are the foundation for all subsequent analyses within the pipeline consisting of the following steps:

1. **Field of View (FOV)-Level Clustering:** The first step involves clustering at the Field of View (FOV) level. This step groups cells within each FOV based on their marker expression profiles, visualized here through UMAP plots that display clusters in a two-dimensional space, showing the relationship between different cell types.

2. **Centroid-Based Cell Typing:** Following FOV-level clustering, SpaFlow employs centroid-based cell typing, where each cluster's centroid (median marker expression values) is used to categorize cells. This step helps in identifying and annotating cell types across the dataset.

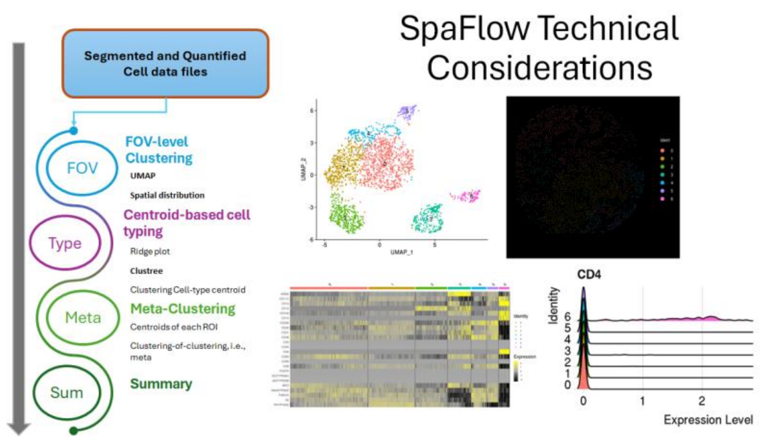

sFig. 1. Overview of the SpaFlow pipeline applied to ovarian TMA cores, starting from segmented and quantified cell data files to the final meta-clustering results.

3. **Meta-Clustering:** The SpaFlow then performs meta-clustering, where clusters across multiple FOVs are further condensed into meta-clusters. This approach facilitates the creation of a consistent set of clusters across large datasets, which is particularly useful when dealing with numerous images or ROIs. The resulting meta-clusters are visualized and analyzed to identify biologically relevant cell populations.

4. **Summary and Reporting:** The final step involves summarizing the results, which includes generating heatmaps and expression level distributions. These summaries provide insights into the cell populations identified, such as the expression levels of key markers like CD4 across different clusters.

Illustrated as supplementary Fig. 2, our meta-clustering approach is designed to combine different FOVs, e.g., multiple cores from a TMA, into a consistent cohort analysis. This method begins with the selection of individual cores from the TMA, each representing a distinct FOV. These cores are stained, imaged, and processed to generate segmented and quantified cell data. Each FOV is initially analyzed independently, with clustering based on specific marker expression, allowing for localized insights into the cellular composition within each core. To ensure consistency and comparability across the entire cohort, the centroid (median marker expression values) of each cluster within each FOV is calculated and used as a representative for merging and re-clustering the data from all FOVs. This step produces "meta-clusters" that represent common cell populations across different cores, providing a consistent framework for cohort-wide analysis. The meta-clusters are further analyzed and visualized, revealing comprehensive views of cell populations across the cohort, with heatmaps illustrating marker expression patterns and network plots demonstrating the relationships and interactions between different cell populations. This integrated analysis facilitates the identification of biologically meaningful patterns and relationships, critical for understanding the tumor microenvironment or other tissue characteristics. By employing this meta-clustering approach, we achieve a robust and scalable method for analyzing large cohorts of FOVs with comparable cell typing solutions.

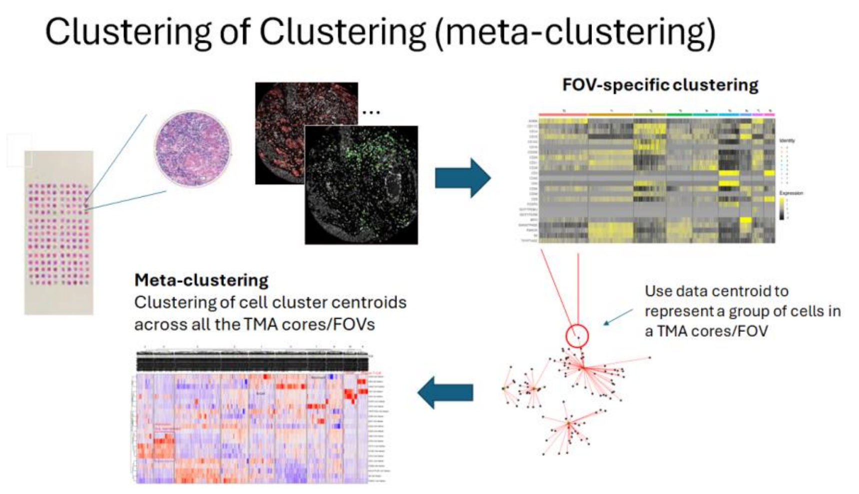

sFig. 2. Illustration of the meta-clustering approach, consolidating clusters from different FOVs within the TMA into consistent meta-clusters for cohort-wide analysis.

Key Results

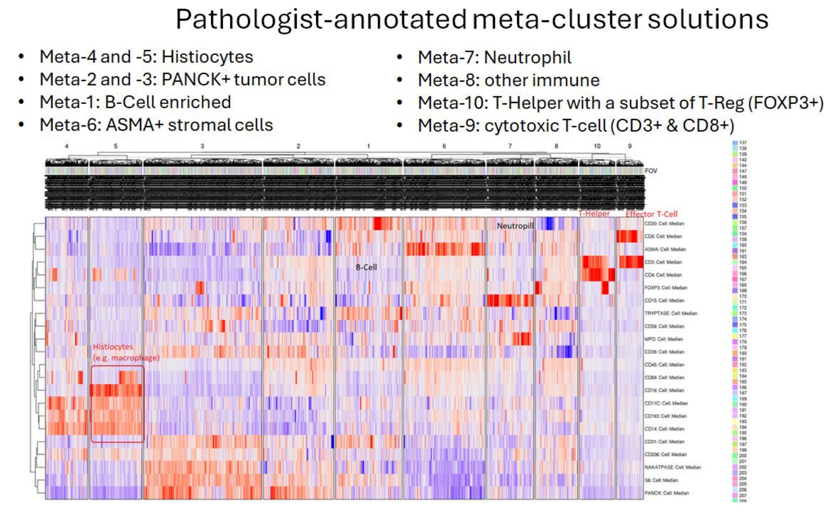

sFig. 3. Heatmap summarizing the pathology annotations of the identified meta-clusters, highlighting distinct immune cell populations within the tumor microenvironment.

Collaborating with a pathologist (R.G.), we summarized the pathology annotations of the meta-cluster solutions identified using SpaFlow (**supplementary Fig. 3**). This figure highlights the distinct cellular populations within the tissue microenvironment, with each meta-cluster corresponding to a unique set of cell types identified across multiple FOVs from the OC TMA, including a few key annotations:

- **Histiocytes (e.g., Macrophages):** This meta-cluster is marked by high expression levels of markers associated with histiocytes, such as CD68 and CD163. These cells play a critical role in immune response and are commonly involved in phagocytosis and antigen presentation.
- **B-Cells:** Another distinct meta-cluster represents B-cells, characterized by the expression of CD20. These cells are key players in the adaptive immune system, responsible for producing antibodies.

- **Neutrophils:** Neutrophils are identified in another meta-cluster, marked by expression of MPO (Myeloperoxidase) and other granulocyte markers. Neutrophils are the first responders in acute inflammation and are crucial for innate immune defense.
- **T-Helper Cells:** This meta-cluster includes T-helper cells, characterized by markers like CD4. These cells are essential for orchestrating the immune response, particularly by helping other immune cells function effectively.
- **Effector T-Cells:** The heatmap also identifies a meta-cluster of effector T-cells, including cytotoxic T cells (CD8+) that are pivotal in targeting and killing infected or cancerous cells.

The heatmap provides a comprehensive view of the expression profiles across different meta-clusters, offering insights into the cellular composition and immune landscape within the tissue samples. The pathology annotations align with known cell lineage markers, validating the meta-cluster solutions identified by SpaFlow. Shown as selective examples in supplementary Fig. 4, we present a FOV of key meta-clusters identified using SpaFlow, specifically meta-cluster 9, representing tumor-infiltrating lymphocytes (TILs) with co-expressed CD3 and CD8, and meta-clusters 2 and 3, which correspond to tumor cells with positive PanCK and variable NAKATPase signals.

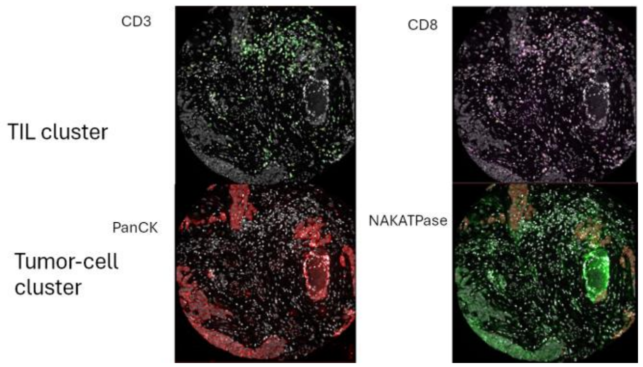

sFig. 4. Selected examples of meta-cluster annotated by the pathologist.

Following our analysis and identification of TIL populations (meta-cluster 9), we conducted a focused validation to compare the TIL cell counts identified through SpaFlow with CD8+ TIL evaluations based on immunohistochemistry (IHC). The validation aimed to assess the concordance between our meta-clustering results and traditional pathological assessments. The boxplot illustrates the relationship between the TIL cell counts within meta-cluster 9 and the pathologist's CD8+ TIL evaluations (score 0 indicates the absence of TILs as per IHC evaluation. Score 1-3 represents weak, intermediate and strong TIL signals). Shown as **supplementary Fig. 5**, this evaluation summarized a positive correlation between the pathologist's CD8+ IHC scores and the TIL cell counts in meta-cluster 9, reflecting the increasing presence of TILs as assessed by IHC. The high concordance between the two methods validates the robustness of SpaFlow's meta-clustering approach, confirming its capability to accurately identify and quantify biologically relevant immune cell populations within the tumor microenvironment.

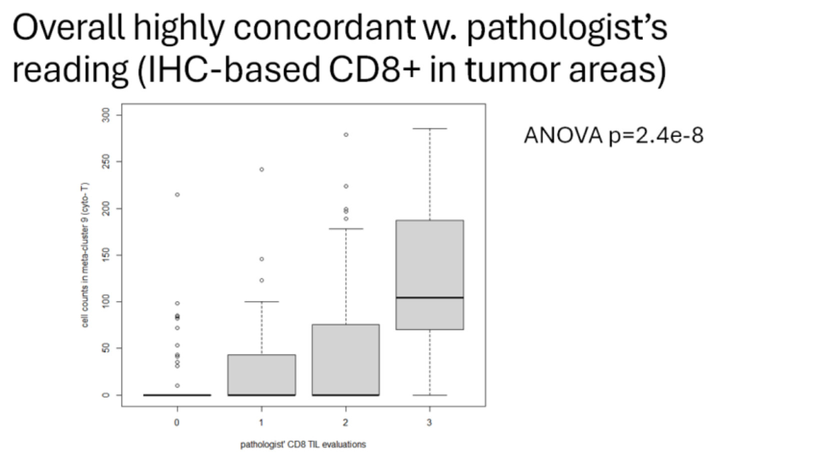

sFig. 5. validation comparing TIL counts of meta-9 cluster cells with CD8 IHC-based evaluations, highlighting the accuracy and reliability of SpaFlow in identifying immune cell populations within the ovarian tumor microenvironment.

Section-5: Use Case 2. Reproducibility Assessment of SpaFlow in Serial Tonsil Sections

Study motivations

In Use Case 2, we focused on assessing the reproducibility of SpaFlow in analyzing serial sections of tonsil tissue. Each section contained 20 Fields of View (FOVs) that were physically adjacent, allowing us to test whether SpaFlow could consistently identify and characterize cellular populations across different but closely related tissue slices. **Supplementary Fig. 6** displays the mapped FOVs across the tonsil sections, with each numbered square representing a distinct FOV. By comparing the data from these adjacent FOVs, we aim to validate the reproducibility of SpaFlow's meta-clustering approach.

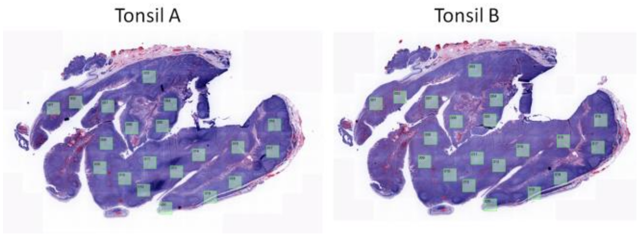

sFig. 6. Overview of 20 mapped FOVs across two serial sections of tonsil tissue, with each numbered square representing a distinct FOV.

Analytical steps and Key Results

Specifically, we examine whether the same cell populations, such as immune cells, are identified consistently across these physically adjacent regions in the serial sections. In the analysis of the tonsil replicates, labeled Tonsil A and Tonsil B, we applied the SpaFlow pipeline to assess the reproducibility of our clustering results. **Supplementary Fig. 7** displays the UMAP plots and spatial visualizations of a selected Field of View (FOV-14) from both tonsil sections. The UMAP plots on the left show the clustering results for Tonsil A and Tonsil B. Each cluster is represented by a distinct color, illustrating the distribution of cell populations within the FOV. The spatial plots on the right provide a visualization of these clusters within the tissue context, with the same color scheme applied to the spatial coordinates of the cells.

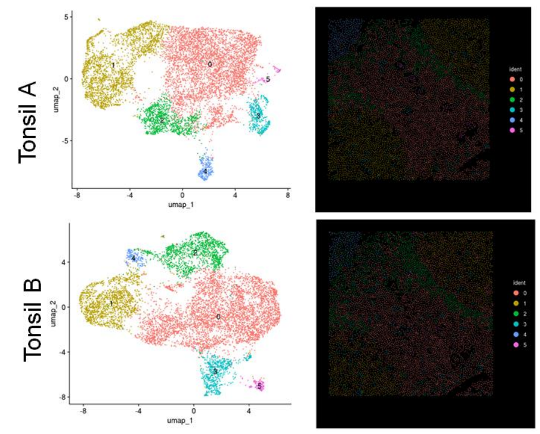

sFig. 7. UMAP plots and spatial visualizations of FOV-14 from Tonsil A and Tonsil B, showing high concordance in the clustering of cell populations identified by SpaFlow.

Starting with the tonsil sections, we mapped and analyzed each FOV independently, applying the SpaFlow pipeline to identify key cell populations through meta-clustering. These meta-clusters represent distinct and interpretable cell populations within the tonsil tissue, as visualized in the heatmap in **Supplementary Fig. 8A**. For example, **meta-1:** Likely represents macrophages, as indicated by high expression levels of markers such as CD68 and CD163, which are typically associated with macrophage populations. **Meta-4:** This meta-cluster is likely composed of B-cells, which are suggested by the expression of CD20, a well-known B-cell marker. **Meta-5:** Represents T-helper cells, characterized by markers like CD4 and CD3. The results demonstrated high concordance between the replicates, e.g. FOV-14 showed consistent clustering and spatial distribution of the identified cell types across both tonsil samples (Tonsil A and Tonsil B), shown in S**upplementary Fig. 8B and 8C**.

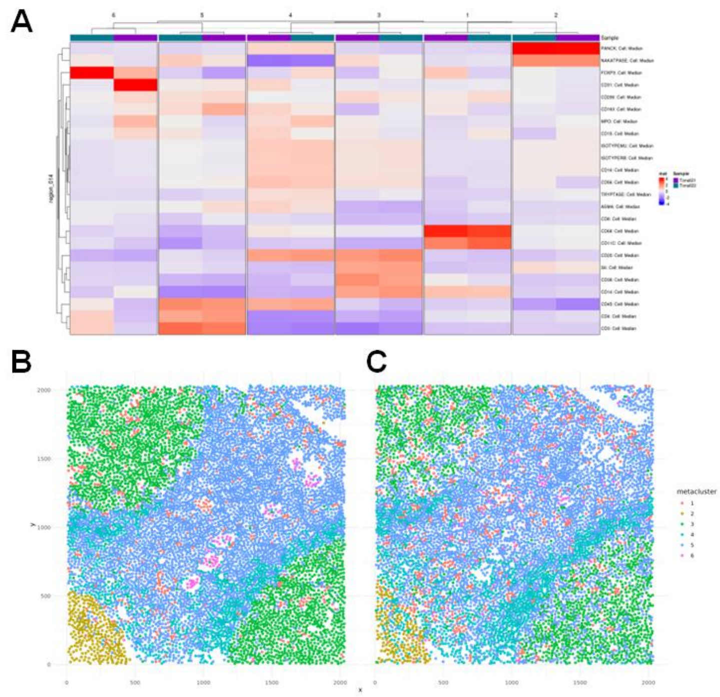

sFig. 8. Meta-cluster solution and SpaFlow solutions' concordance. **(A)** The upper heatmap showing the median expression levels of various markers across the six major meta-clusters identified in the tonsil samples, with consistent profiles observed between Tonsil A and Tonsil B (Bottom plots).

We also performed a quantitative evaluation of concordance by calculating the Spearman correlation of cellular compositions across corresponding regions in the two samples. The high correlation values, with an average of 0.852 and a median of 0.886 for FOV-14, underscored the reproducibility and reliability of SpaFlow in capturing true biological patterns within complex tissues.

The serial section analysis also allowed us to demonstrate SpaFlow's ability to aid in QC by identifying large image artifacts such as nonspecific staining. These artifacts can cause entire clusters to form around an area of nonspecific staining. An example can be seen in Supplementary Figure 9, where in the serial section Tonsil 1, nonspecific staining of rabbit IgG (Isotype RB) has caused a meta-cluster unique to Tonsil 1 highly expressing Isotype RB, which is evident in the heatmap. In these cases, SpaFlow clusters may be used as a first-iteration QC to identify which marker caused the artifact. Users may decide to exclude the problematic marker from further analysis by removing it from the "markers" parameter in the Nextflow configuration file. Alternatively, the user may exclude affected cells by using the "filter_column" parameter in the Nextflow configuration file. This feature allows users to specify the name of a custom column in the quantification file which contains "1" to include a cell segment, or "0" to exclude it. Once these measures have been taken, users may SpaFlow again and interpret the clusters in the context of cell phenotypes.

Overall, these findings confirm that SpaFlow provides robust and consistent results across different tissue sections, making it a valuable tool for spatial biology studies, especially when accurate and reproducible analysis of tissue microenvironments is critical.

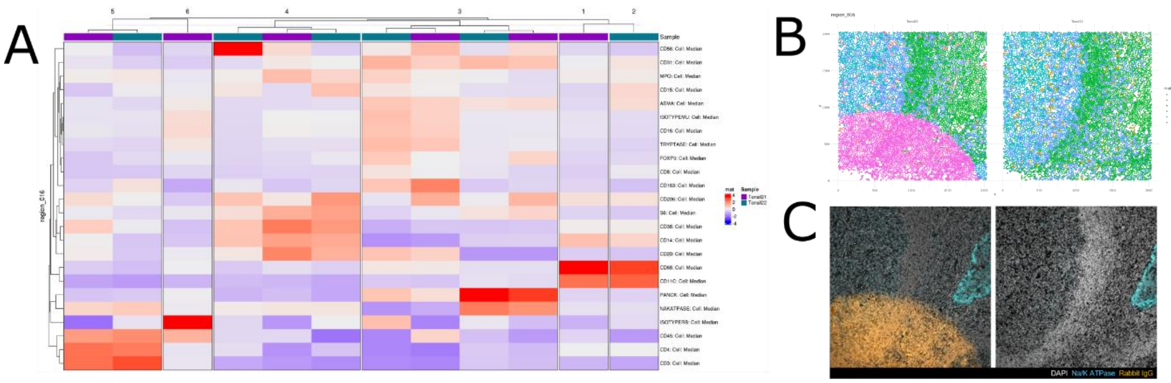

sFig 9. Large nonspecific staining artifact identified by SpaFlow meta-cluster solution. (A) Heatmap showing meta-cluster 6, unique to Tonsil A, which is highly enriched for Rabbit IgG, a nonspecific marker. (B) Spatial distribution of meta-clusters, showing meta-cluster 6 only occurring in Tonsil A (C) MxIF staining for DAPI (white), Rabbit IgG (orange), and Na/K ATPase (cyan), showing the large nonspecific staining artifact in Tonsil A.

Section-6: Use Case 3. Assessment of SpaFlow with Alternate Panel Designs with Oropharyngeal Squamous Cell Carcinoma Imaging Mass Cytometry Dataset

Study motivations

The goal of Use Case 3 was to demonstrate SpaFlow's flexibility across different panel designs. Two of the main advantages of unsupervised clustering approaches over supervised classification methods are panel flexibility and low barrier to analysis. Prediction of cell types via pretrained models requires the unlabeled dataset to contain all of the markers that were present in the model's training dataset. However, MxIF marker panel designs may vary between experiments, meaning that markers present in the training dataset may be missing from the unlabeled dataset, or that potentially informative markers in the unlabeled dataset cannot be leveraged for cell type prediction. Training new supervised classification models can solve this, but training requires time-consuming expert annotation, creating a high barrier to analysis. Unsupervised clustering methods such as those included in SpaFlow can alleviate these problems. Unsupervised clustering can be performed on any combination of markers. Classification based on cluster-wide marker expression can provide biologically meaningful phenotype results, but if higher accuracy is required, phenotypes from clustering analysis may be used as a first-pass classification to expedite the annotation process for further iterations with supervised methods.

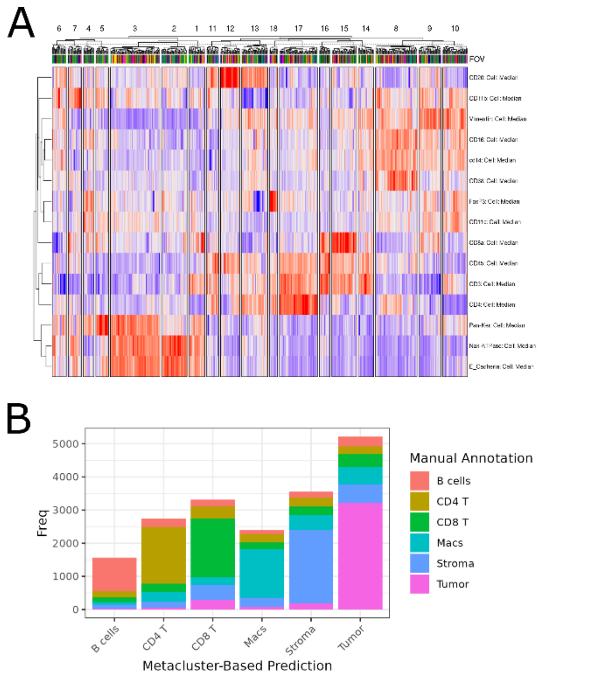

sFig 10. Validation of meta-clusters with manual annotations. (A) Meta-cluster heatmap used to classify cells based on overall marker expression. (B) Composition of each meta-cluster phenotype group compared to expert annotations.

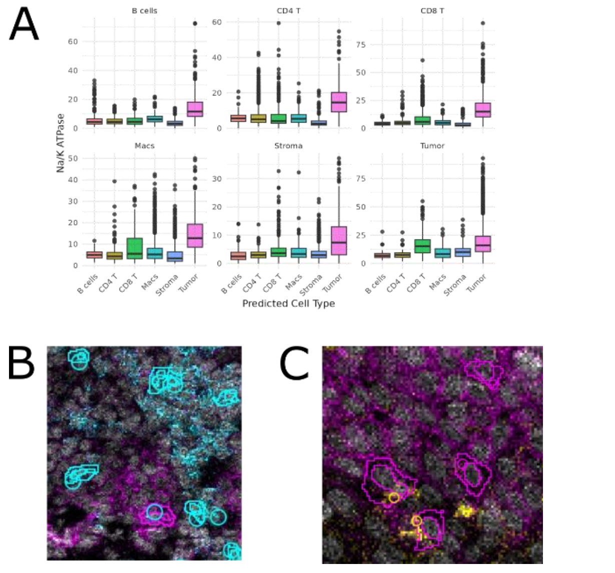

sFig 11. (A) Misclassified cells show higher expression of markers for misclassified cell type. Across all annotated cell types, cells misclassified as Tumor showed higher expression of Na/K ATPase. Example of B cell (B) and macrophage (C) misclassified as tumor cells due to surrounding tumor marker expression. Cyan stain denotes CD20 (B cell marker), cyan cell outlines denote B cell predictions. Yellow stain and circles denote CD68 expression and macrophage annotations, respectively. Cyan circles denote B cell annotations, magenta stain denotes Na/K ATPase (tumor marker), magenta outline denotes tumor cell prediction.

Analytical steps and Key Results

To demonstrate SpaFlow's flexibility across panel designs, as well as diverse tissue types and imaging platforms, we analyzed a dataset of approximately 20,000 cells in 64 ROIs from oropharyngeal squamous cell carcinoma (OPSCC) patients (Bartemes et al.) generated on the Hyperion multiplex imaging mass cytometry platform (Standard Biotools). The OPSCC dataset introduces several markers which are absent in the ovarian dataset from Use Case 1, including vimentin, CD11b, CD45RA, and CD45RO. Conversely, the OPSCC panel does not include several markers available in the ovarian dataset, including the isotype controls, tryptase, and MPO. To verify the clustering results, manual phenotype annotations were made available for all cells included in the analysis. We employed the analytical steps described in Section 4 to generate meta-clusters across all ROIs for classification.

From 18 meta-clusters, cells were merged into six cell phenotypes: B cells, CD4 T cells, CD8 T cells, macrophages, stromal cells, and tumor cells, based on the overall meta-cluster marker expression specific to cell lineages (Supplementary Figure 10A). The manual annotations

enriched all predicted phenotype classes in the resulting meta-cluster solutions (Supplementary Figure 10B). We further examined the misclassified cells to determine possible causes, focusing on misclassified cells that showed higher expression of markers characteristic of the misclassified cell type (Supplementary Figure 11A). When the SpaFlow-led results were reviewed visually, incorrect predictions tended to be single cells of a given phenotype surrounded by cells of another type, with so-called “bleed-over” signals causing misclassification by the unsupervised algorithm (Supplementary Figure 11B-C). In other cases, the disparity prompted further examination of the cell in question, and the SpaFlow-generated result was accepted over the original annotation. This demonstrates the utility of a data-driven approach in supporting human annotators. Despite the nuanced misclassification examples, the SpaFlow and QuPath visualizations can iteratively improve the cell phenotyping results by presenting initial unsupervised clustering to be further refined through human annotations, and to exclude regions with quality artifacts, eventually leading to more accurate supervised classification models.
